# Psychological functioning in pregnant women who experienced complex trauma

**DOI:** 10.3389/fgwh.2025.1611034

**Published:** 2025-07-31

**Authors:** Geneviève Lapointe, Christine Drouin-Maziade, Julia Garon-Bissonnette, Florence Bordeleau, Roxanne Lemieux, Nicolas Berthelot

**Affiliations:** ^1^ Department of Psychology, Université de Sherbrooke, Sherbrooke, QC, Canada; ^2^ Centre D'études Interdisciplinaires sur le Développement de L'enfant et la Famille, Trois-Rivières, QC, Canada; ^3^ Interdisciplinary Research Centre on Intimate Relationship Problems and Sexual Abuse (CRIPCAS), Montreal, ON, Canada; ^4^ Groupe de Recherche et d’intervention Auprès de l’enfant Vulnérable et Négligé, Trois-Rivières, QC, Canada; ^5^ Department of Nursing Sciences, Université du Québec à Trois-Rivières, Trois-Rivières, QC, Canada; ^6^ Department of Psychology and Human Development, Peabody College, Vanderbilt University, Nashville, TN, United States; ^7^ Cervo Brain Research Center, Quebec, QC, Canada

**Keywords:** developmental trauma, complex PTSD, pregnancy, mental health, childhood maltreatment

## Abstract

**Introduction:**

The concept of complex trauma, which has been operationalized by the diagnosis of developmental trauma disorder (DTD) in children and adolescents, may contribute to our understanding of the large interindividual variability in maternal health and functioning among pregnant women who experienced childhood maltreatment. The study examines whether three groups of pregnant women (one including women who experienced childhood maltreatment and suffered from DTD, a second including women who experienced childhood maltreatment but did not suffer from DTD, and a third group including women who did not report experiencing childhood maltreatment) differ on mental health and functioning during the prenatal period. Several markers associated with the intergenerational trajectories of childhood maltreatment were examined: severity of PTSD symptoms, quality of prenatal attachment, perception of maternal competence, reflective functioning, disruptions in mentalization of trauma and adverse relationships, intimate partner violence, and mental health disorders.

**Methods:**

The study includes 373 pregnant women who participated in a comprehensive diagnostic assessment of current and lifetime psychiatric disorders conducted by two blinded and independent clinical psychologists. The women also completed self-report measures of symptoms and functioning.

**Results:**

Women with DTD (*n* = 26) had more severe symptoms of PTSD, lower quality of prenatal attachment to the fetus, lower perceptions of maternal competence, less curiosity about mental states, and more severe disruptions in mentalizing trauma and adverse relationships than women who experienced childhood maltreatment but never met the diagnostic criteria for a DTD (*n* = 99) and women not exposed to childhood maltreatment (*n* = 248). In contrast, women who experienced childhood maltreatment but did not develop a DTD did not differ from women not exposed to maltreatment on all domains except the level of disruptions in mentalizing trauma and adverse relationships. Women who had a DTD in childhood or adolescence also had an 18.5-fold and 25.4-fold increased risk of having a mental health disorder during pregnancy compared, respectively, to women who had experienced maltreatment without DTD and women who had not experienced maltreatment. Persistent complex trauma, defined as the presence of a diagnosis of Complex PTSD during pregnancy, was present in over a third (34.6%) of women with DTD.

## Introduction

Childhood maltreatment, under the form of abuse or neglect before the age of 18 years, is a pervasive phenomenon that affects approximately one-third of the population (1). Its adverse consequences are evident in both the short and long terms (2–4). Several meta-analyses and systematic reviews showed strong associations between childhood maltreatment and adult psychopathology, including internalized disorders [e.g., anxiety, depression (5, 6)], post-traumatic stress disorder [PTSD (7, 8)], externalized disorders [e.g., ADHD (9)], major psychiatric disorders [e.g., psychosis and bipolar disorders (10, 11)], personality disorders (12), and substance use (13).

Childhood maltreatment also represents a significant risk factor for women's health and functioning during pregnancy (14, 15), which may partly explain its well-documented intergenerational effects (16–18). Accordingly, childhood maltreatment is rather frequent in community samples of pregnant women — concerning up to 35% of women from low-risk samples (19) — and associated with higher risks of depression (20, 21), PTSD (22, 23), dissociation (24, 25), anxiety (26, 27), and personality disorders (28). A dose-response association has also been observed between the severity of exposure to childhood maltreatment and the severity (29) and complexity of psychiatric symptoms in pregnant women (19, 23). The association between childhood maltreatment and psychopathology in pregnant women holds for different types of abuse and neglect (30, 31) and appears consistent across cultures (32).

This increased risk of psychological problems during pregnancy in women with a history of maltreatment has implications for maternal and marital functioning during and after pregnancy. For instance, previous research showed that childhood maltreatment was indirectly associated, through the severity of psychiatric symptoms, with lower maternal confidence (33, 34) and poorer quality of antenatal attachment (33, 35–37) in pregnant women. Prenatal psychiatric symptoms were also shown to be prospectively associated with increased bonding difficulties (38), less sensitive parenting behaviors (39), and poorer infant socioemotional development (40–44).

In addition to increased psychological distress during pregnancy, exposure to abuse or neglect during childhood may preclude pregnant women from accessing protective factors that could mitigate the intergenerational effects of maltreatment and mental disorders, including the experience of positive and nurturing relationships and the development of effective mentalizing skills. Firstly, research has shown that pregnant women who have experienced childhood maltreatment are more at risk of being in a violent marital relationship than women without such a history (45, 46): the more severe the childhood maltreatment, the more severe the intimate partner violence (47). In turn, this elevated risk of intimate partner violence has been associated with higher levels of parental stress (48) and reduced maternal sensitivity (49). Secondly, childhood maltreatment has been linked to various forms of impairments in mentalizing (50, 51), defined as the capacity to understand behaviors in terms of intentional mental states (52). For instance, childhood maltreatment was positively associated with a lack of curiosity about one's and others’ mental states, as assessed using the Reflective Functioning Questionnaire (53, 54). Poor reflective functioning also explained a large part of the association between maternal history of childhood maltreatment and poor psychological functioning during pregnancy (55) as well as developmental delays in their offspring (56). Furthermore, trauma-specific reflective functioning, defined as the ability to mentalize more specifically about past experiences of abuse and neglect and adverse relationships (57), was demonstrated as a robust predictor of maternal functioning in samples of mothers who have experienced childhood maltreatment. Indeed, the severity of disruptions in mentalizing trauma or adverse relationships has been associated with the severity of psychiatric symptoms (i.e., anxiety, depressive, dissociative symptoms and personality dysfunction) and intimate partner violence (57, 58) in pregnant women. In contrast, more complex abilities to mentalize experiences of childhood maltreatment were associated with a positive investment regarding motherhood and with the quality of the parental couple functioning during pregnancy (59), the quality of the mother-child attachment relationship (60), maternal insightfulness regarding infants' emotional needs (61), and a lower likelihood of intergenerational cycles of childhood sexual abuse (62).

### Complex trauma and functioning in pregnant women

Although childhood maltreatment is a major risk factor for long-term health and adjustment, poor outcomes are not inevitable. Indeed, most pregnant women who have experienced childhood abuse or neglect would be resilient (58). This immense interindividual variability observed in mental health outcomes following maltreatment remains largely misunderstood. Recent developments in the field suggest that *complex trauma* may account for some of this heterogeneity (63). Complex trauma is characterized by the co-occurrence of 1- traumatic events (abuse, neglect, or witnessing domestic violence) occurring during childhood in the context of relationships with attachment figures or through repeated or prolonged disruptions with caregivers, that caused direct harm and compromised the child's physical and psychological development; and 2- invasive sequelae affecting different domains including cognitive, affective, relational, and behavioral functioning (64, 65).

Complex trauma was operationalized in two ways in clinical research and practice. First, in children, complex trauma is mainly assessed through the diagnosis of *Developmental Trauma Disorder* [DTD (66)]. DTD refers to children or adolescents who have been exposed to repeated and severe episodes of traumatic *interpersonal victimization,* as well as traumatic *disruption of protective caregiving* due to primary caregiver changes, separation, or impairment, or emotional abuse (Criterion A), and who developed pervasive symptoms (at least 6 months) impacting functioning categorized into three domains of biopsychosocial dysregulation (67). The first domain, *affective/somatic dysregulation* (Criterion B), includes four symptoms that reflect maladaptive emotional processing (e.g., alexithymia), extreme emotional dysregulation, somatic dysregulation, and dissociation. The second domain, *cognitive/behavioral dysregulation* (Criterion C), contains five symptoms that reflect problems in information processing and related difficulties with behavioral activation and self-control. These include impulsivity, risky behaviors, confrontational actions, self-harm, dysfunctional self-soothing, challenges with initiating or completing goal-directed behavior, and preoccupation with potential threats. The third domain, *self and interpersonal dysregulation* (Criterion D), consists of symptoms that involve difficulties with self-concept (perceiving oneself as permanently broken and enmeshed/overly permeable psychological boundaries), and dysregulation in relationships, under the forms of avoidant or aggressive patterns of engagement, expectations of betrayal, disorganized attachment and either excessive or deficient empathy. Despite it is not yet included in most used nomenclatures of mental health disorders, the diagnosis of DTD has strong empirical support (68).

Second, in adults, complex trauma has been operationalized through the ICD-11 diagnosis of complex post-traumatic stress disorder [CPTSD, see p. 344 (69)]. CPTSD is a condition that arises from prolonged exposure to an extremely threatening or horrific event or series of events. It combines the usual symptoms of PTSD (re-experiencing the traumatic event, avoidance of trauma-related reminders, and an increased sense of threat) with significant and persistent difficulties regulating emotions, a shameful and negative sense of self, and disturbances in relationships with others.

Despite previous suggestions that DTD may have a long-lasting course, including complex psychological symptoms in adulthood (70), no prior studies to our knowledge have examined whether DTD had persisting effects on pregnant women's health, well-being and functioning. Accordingly, it remains unclear whether women with histories of DTD exhibit poorer outcomes compared to those who experienced childhood maltreatment without developing a DTD, and whether these two groups demonstrate altered psychological functioning in comparison to pregnant women who did not experience childhood maltreatment.

### Current study

The current study aims to assess the frequency of a probable history of DTD in a community sample of pregnant women and examines whether women who experienced DTD during childhood or adolescence differ from pregnant women who experienced childhood maltreatment without developing a DTD and from those with no history of childhood maltreatment. The comparison focuses on several markers of mental health and functioning that have been shown to be key mechanisms in the intergenerational trajectories of maltreatment. These include PTSD symptoms severity, quality of prenatal attachment, perceived maternal competence, impairments in mentalizing, severity of intimate partner violence and prevalence of mental health disorders. We hypothesized that women who experienced childhood maltreatment, regardless of whether they developed a DTD, would exhibit poorer functioning across all risk indicators compared to those without a history of childhood maltreatment. However, we expected that women with a history of DTD would demonstrate more severe impairments in both maternal and psychological functioning than those who experienced maltreatment without developing a DTD. Finally, we also examined the persistence of complex trauma over time by assessing the frequency of women with both a probable diagnosis of DTD in childhood/adolescence and a diagnosis of CPTSD during pregnancy.

## Methods

### Participants and procedure

A sample of 377 pregnant women aged between 18 and 43 years old (*M_age_* = 29.17, *SD* = 4.46) from the general population was recruited at pregnancy-related medical appointments in Quebec, Canada, as part of a larger longitudinal study on child maltreatment and the transition to parenthood. Data collection took place between 2015 and 2022. Exclusion criteria for participation in the study were being under 18 years of age, having a psychotic disorder or an intellectual disability, expecting a multiple birth, or expecting a child who is suspected of having a neurodevelopmental congenital disorder. Four participants were excluded (one did not complete the Childhood Trauma Questionnaire, two did not complete the SCID interview, and one had a psychotic disorder), resulting in a final sample of 373 participants. Among them, 33.5% (*n* = 125) experienced at least one form of childhood maltreatment based on the cut-offs of the Childhood Trauma Questionnaire (outlined below) and were categorized as having experienced childhood maltreatment. Participants were predominantly white (*n* = 346, 97.2%). Half participants were above the Quebec median annual family income (*n* = 179, 49.7%). Sociodemographic data are shown in Table 1. Most participants were employed or on a preventive leave (88.5%), had post-secondary education (92%) and were in a relationship with the father of the child (96.5%). All women who participated in the researched were given CAN $10 for their time.

**Table 1:** Sociodemographic characteristics of participants without childhood maltreatment, and participants exposed to childhood maltreatment without DTD and with DTD.

Sociodemographic characteristics	Total *n* = 373	Controls *n* = 248	CM without DTD *n* = 99	CM with DTD *n* = 26	Group differences F or *x*^2^	Significant contrasts
Age, *M* (*SD*), [range]	29.2 (4.5) [18 to 43]	29.2 (4.3) [18 to 43]	29.7 (4.6) [21 to 42]	26.8 (5.1) [19 to 40]	*F* (2,366) = 4.31*	C > DTD*, CM without DTD > DTD*
Ethnicity, *N* (%)^a^						
White	346 (97.2)	230 (97.4)	93 (97.9)	23 (92)	*x*^2^ = 12.68	N.S.
Black	4 (1.2)	3 (1.3)	0	1 (4)		
Latina	3 (.8)	3 (1.3)	0	0		
Other	3 (.8)	0 (0)	2 (2.1)	1 (4)		
Annual Familial Income (CAD), *N* (%)^b^						
34,999$ or less	32 (8.9)	12 (5)	12 (12.9)	8 (32)	*x^2^ (2)* *=* 26.44***	C > CM without DTD*,DTD*** CM without DTD > DTD*
35,000$ - 84,999$	149 (41.4)	101 (41.7)	36 (38.7)	12 (48)		
85,000$ or more	179 (49.7)	129 (53.3)	45 (48.4)	5 (20)		
Educational status, *N* (%)						
Primary or high school	30 (8)	10 (4)	11 (11.1)	9 (34.6)	*x^2^ (4)* *=* 40.33***	C > CM without DTD***, DTD*** CM without DTD > DTD**
College or professional	181 (48.6)	113 (45.6)	58 (58.6)	10 (38.5)		
University	162 (43.4)	125 (50.4)	30 (30.3)	7 (26.9)		
Marital status, *N* (%)^a^						
Married or cohabiting	360 (96.5)	240 (96.8)	95 (96)	25 (96.2)	*x^2^ (2)* *=* .15	N.S.
Single parent	13 (3.5)	8 (3.2)	4 (4)	1 (3.8)		

C, controls; CM, childhood maltreatment; DTD, developmental trauma disorder; N.S., non significant; PTSD, post-traumatic stress disorder.

^a^ Fisher exact test was used when some cells had less than 5 participants.

^b^ The low-income cut-off by census metropolitan area of Quebec in 2018 for a family with 1 child is 34,295$ and the median income is of 84,165$.

* *p* < .05. ** *p* < .01. *** *p* < .001.

Data collection included two activities. First, participants were asked to complete a series of questionnaires online or in paper format at home. Second, participants were met in our facilities, at home, or by videoconference to complete structured clinical interviews assessing current and lifetime psychiatric history during pregnancy or the first months postpartum. The study was approved by our Universities and Regional Health Centers ethics committees.

### Measures

#### PTSD

Symptoms of PTSD during the last month were measured with the French version (71) of the PTSD Checklist for DSM-5 [PCL-5 (72)]. The instrument includes 20 items based on the DSM-5 PTSD diagnostic criteria and used a 5-point Likert scale ranging from 0 (not at all) to 4 (extremely). Scores vary between 0 and 80, with high scores reflecting more intense symptomatology. The French version has good internal consistency (Cronbach alpha of .94), good test-retest reliability (*r* = .89), and good convergent validity [*r* = .82, *p* < 0.001 (71)]. The Cronbach's alpha for the PCL-5 in this study was of .93.

#### Maternal functioning

This dimension was assessed through two complementary constructs: maternal antenatal attachment and perception of parental competence. First, antenatal attachment was measured using the Quality subscale of the Maternal Antenatal Attachment Scales [MAAS (73)]. This scale includes 11 items using a variable 5-point Likert scale. A high score indicates a strong emotional attachment to the fetus. The MAAS has good psychometric properties (73) and the Cronbach's alpha for the quality of attachment subscale of the MAAS in this study was of .75.

Second, perception of parental competence was assessed using the Maternal Confidence Questionnaire [MCQ (74)]. The instrument includes 14 items and uses a 5-point Likert scale ranging from 1 (never) to 5 (very often). A high score indicates a perception of oneself as having good parenting skills. A literature review revealed good construct validity and internal consistency of the MCQ in over twenty studies [mean *α* = .89 (75)]. The Cronbach's alpha for the MCQ in this study was of .88.

#### Mentalizing

Two complementary forms of mentalizing impairments were assessed: hypomentalization and disruptions in mentalizing trauma and adverse relationships. First, the Uncertainty scale of the French version (76) of the Reflective Functioning Questionnaire (RFQ-8) was used to capture *hypomentalization*, i.e., the tendency to show a complete lack of knowledge about mental states and to rely mainly on concrete thinking (76). Responses to the 6 items of the Uncertainty scale are rated on a 7-point Likert scale ranging from 1 (*completely disagree*) to 7 (*completely agree*). Scores are subsequently rescored using a median-scoring method: original responses (from 1 to 7) are scored 0, 0, 0, 0, 1, 2, 3, meaning that low to moderate agreement with the item (e.g., I don't always know why I do what I do) reflects adequate understanding that mental states are opaque, while high levels of agreement reveal a complete lack of knowledge about one's own and others' mental states. The original English version of the instrument (77) and the French version showed good psychometric properties (76, 77). The Cronbach's alpha for the Uncertainty scale of the RFQ in this study was of .74.

Second, *disruptions in mentalizing trauma and adverse relationships* (i.e., indications that the respondent is not able to maintain coherent thinking when remembering or discussing negative experiences and/or indices of definite distortions in the perception of the impact of past abuse and neglect and adverse relationships on the self, mental states, and behaviors) were assessed using the original French version of the Failure to Mentalize Trauma Questionnaire [FMTQ (57)]. The questionnaire invites participants to respond to its 39 items when thinking of previous interpersonal experiences during which they felt intense negative emotions, such as betrayal, hurt, abandonment, exploitation, disrespect, fear, or being overwhelmed. Responses are rated on a 5-point Likert scale from 0 (*completely disagree*) to 4 (*completely agree*). Higher scores reflect higher presence of disruptions in mentalizing trauma and adverse relationships. The global score is obtained by adding the mean score at each of the seven subscales of the instrument. The FMTQ has good psychometric properties (57). The Cronbach's alpha for the FMTQ in this study was of .93.

#### Intimate partner violence

A 24-item version (78) of the Revised Conflict Tactics Scale [CTS-2 (79)] was used to assess psychological and physical violence victimization and perpetration. The 8-point Likert scale measures the frequency of interpersonal violence since the beginning of pregnancy, with responses ranging from 0 (never happened) to 6 (more than 20 times since being pregnant). A score of 7 indicates that the behavior did not occur during the pregnancy but had occurred previously and was recoded 0. The instrument provides separate scores for victimization and perpetration. Higher scores reflect more frequent victimization or perpetration of psychological and/or physical violence. The CTS-2 demonstrated good reliability and validity across various non-clinical samples of adults (79, 80). In the current study, the Cronbach's alphas were .67 for the victimization scale and .71 for the perpetration scale.

#### Childhood maltreatment

Experiences of abuse and neglect were retrospectively assessed using the French version (81) of the Childhood Trauma Questionnaire [CTQ-28 (82)]. The instrument includes 28 items measuring the degree of exposure to five types of maltreatment: physical, sexual, and emotional abuse, physical and emotional neglect. Responses are rated on a 5-point Likert scale ranging from 1 (never true) to 5 (always true). Higher scores indicate more severe exposure to childhood maltreatment. Cut-offs were validated for each subscale: physical abuse ≥8, sexual abuse ≥8, psychological abuse ≥10, physical neglect ≥8, emotional neglect ≥15 (83). Participants were assigned to the group with a history of maltreatment if they reached the cut-off on at least one scale. The Cronbach's alpha for the CTQ-28 in this study was of .94.

#### DSM-5 disorders

A best-estimate diagnostic procedure was conducted to assess both current and lifetime DSM-5 diagnoses. This procedure involved a structured, multi-step approach led by trained doctoral students in psychology. First, participants underwent an initial screening interview inspired by *Essentials of Psychiatric Diagnosis, Revised Edition* (84). This screening included targeted questions designed to identify the potential presence of past or current symptoms characteristic of various psychological disorders. When participants endorsed symptoms indicative of a DSM-5 disorder, the corresponding section of the Structured Clinical Interview for DSM-5 Disorders (SCID-5), either the Research Version (SCID-5-RV) or the Personality Disorders Version (SCID-5-PD), was administered. Interviews were recorded to allow for detailed documentation of participants' life histories and symptomatology. Once all relevant information was transcribed into a secure and anonymized research file, the recordings were permanently deleted. The collected data were then reviewed by an independent panel of two psychologists with expertise in childhood maltreatment and psychiatric diagnosis. These experts conducted a blind consensus evaluation to determine current and lifetime DSM-5 diagnoses. In cases where consensus could not be reached, participants were recontacted for additional clarification to refine the diagnostic assessment.

#### Complex trauma

DTD and CPTSD diagnoses were retrospectively established using a similar structured, multi-step approach. In the first step, two independent psychologists and a doctoral student in clinical psychology developed a coding-grid for DTD based on Ford et al. (85) criteria. The presence of a DTD during childhood or adolescence required two dual conditions. First, the individual must have directly experienced or witnessed repeated and severe episodes of interpersonal violence AND must have faced significant disruptions in the protective role of caregivers, characterized by one or more of the following: repeated changes in primary caregiver, repeated separation from the primary caregiver, and/or exposure to severe and persistent emotional abuse by a caregiver. Second, one had to have presented all three categories of symptoms during childhood or adolescence: affective and physiological dysregulation (≥3 symptoms), attentional and behavioral dysregulation (≥2 symptoms), self and relational dysregulation (≥2 symptoms). A similar grid was developed for CPTSD based on the CIM-11 criteria [see p. 344–348 (69)]. In a second step, DTD and CPTSD were independently coded by a clinical psychologist and a Ph.D student in clinical psychology using all available information in the detailed research notes from the lifetime psychiatric assessment (see previous section) and self-reported measures of maltreatment [including the CTQ-28, but also the Childhood Interpersonal Trauma Inventory Checklist, a novel measure of a wide range of potentially traumatic experiences (86)]. After independent assessment, the raters met to reach a consensus classification for each participant.

#### Socio-demographic information

Sociodemographic information such as age, ethnicity, marital status, and economic status was collected to account for the potentially confounding role of these variables in statistical analyses.

### Data analysis

Data were missing for the following variables: age (1.1%), ethnicity (3.2%), annual familial income (3.2%), education status (0%), marital status (0%), childhood maltreatment (4%), PTSD symptoms (7.2%), antenatal attachment (.8%), maternal confidence (3.5%), hypomentalization (1.9%), disruptions in mentalizing trauma (11.5%), and intimate partner violence (3.5%). Missing data were completely at random (MCAR) for all variables, *x*^2^(301) = 321.38, *p* = .20. Analyses were therefore performed using only complete cases. A total of 35 outliers were winzorized prior to analyses (7 for CTQ-28, 4 for MAAS, 2 for MCQ, 5 for RFQ-8, 5 for CTS-2 (perpetration), and 12 for CTS-2 (victimization).

ANCOVAs controlling for relevant confounding variables were performed to evaluate group differences on all of the seven external variables. Three groups were formed on the basis of participants’ history of maltreatment (yes/no) and history of DTD (yes/no): a control group comprising women without history of childhood maltreatment, a group including women who experienced childhood maltreatment without developing a DTD, and a group comprising women who experienced maltreatment and developed a DTD during childhood or adolescence. When the postulates of analyses of variances were not met for a specific variable, nonparametric ANCOVAs of Quade were used. A logistic regression was also used to evaluate whether women with childhood maltreatment without DTD or with DTD were at increased risk of suffering from a mental health disorder during pregnancy, using women without history of childhood maltreatment as a reference group. Chi-square analyses were finally performed to evaluate whether different mental health disorders were homogeneously distributed among the three groups of pregnant women. To limit the risk of bias, a Bonferroni correction was applied to account for multiple testing and the *p*-values were fixed to *p* < .007 (.05/7 tests) for the ANCOVAs and *p* < .005 (.05/10 tests) for the Chi-square analyses. All the analyses were performed using SPSS version 29.0.

## Results

### Preliminary analyses

In this community sample of pregnant women, 248 (66.49%) did not experience maltreatment, 99 (26.54%) experienced maltreatment but did not develop a DTD, and 26 (6.97%) experienced maltreatment and met the criteria for a DTD during childhood or adolescence. As shown in Table 1, women who experienced childhood maltreatment and developed a DTD during childhood or adolescence were significantly younger, less educated, and had lower income than women without histories of maltreatment and women who experienced childhood maltreatment but did not develop a DTD. Women with histories of maltreatment who did not develop a DTD had lower income and education than women without maltreatment. Further analyses thus controlled for age and education (income was not included as a distinct covariate given its strong correlation with education). Furthermore, since participants who experienced childhood maltreatment but did not develop a DTD had lower CTQ scores (*M* = 45.69, *SD* = 11.82) than participants with DTD (*M* = 52.54, *SD* = 12.69), *t* (116) = −2.50, *p* = .01, *d* = .57), we also performed analyses contrasting these two specific groups by adding the severity of exposure to maltreatment as a covariate. These analyses are reported in Supplementary Table S1.

### Principal analyses

**Figure 1:**
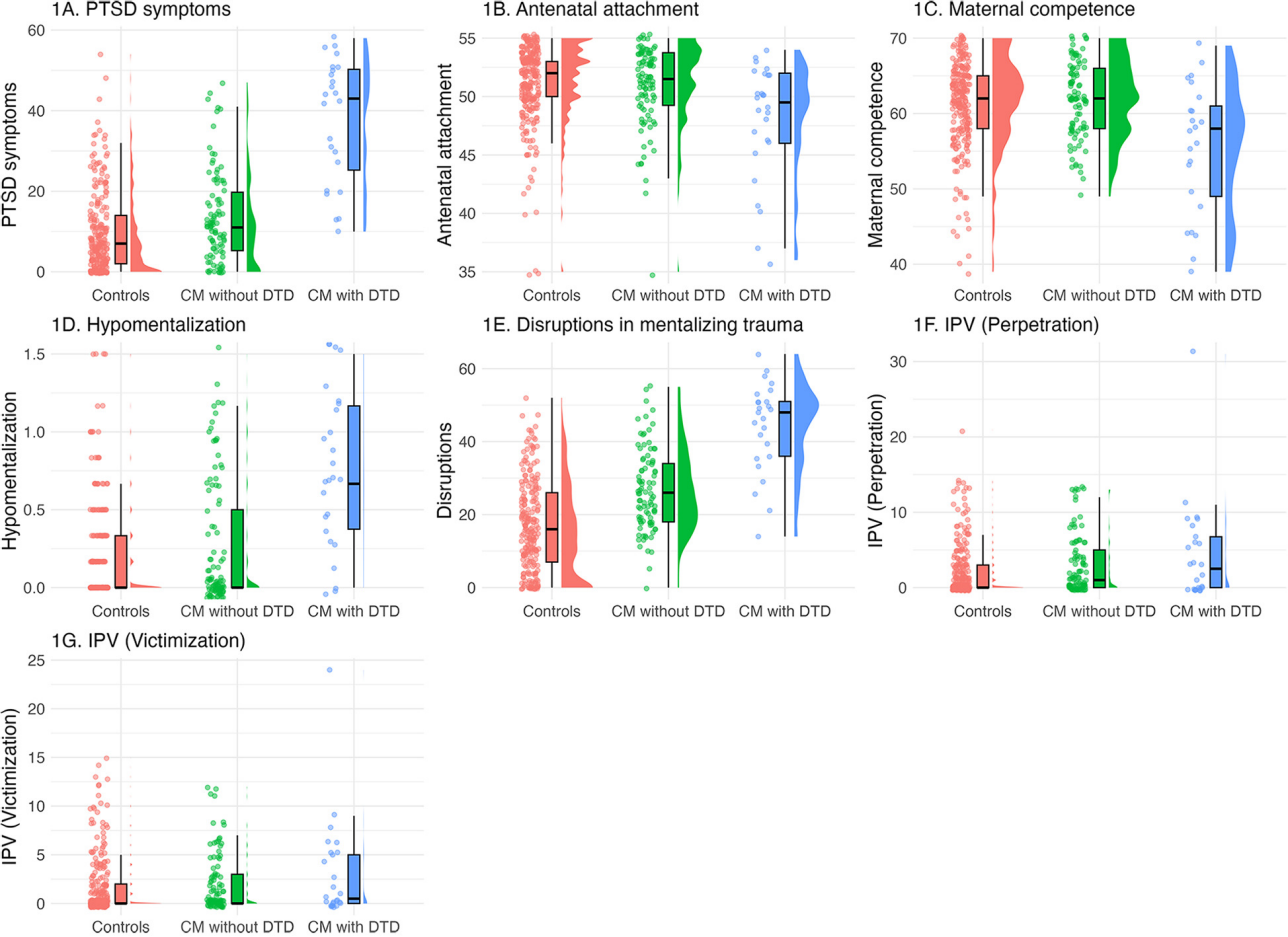
Distributions between participants without childhood maltreatment, and participants exposed to childhood maltreatment without DTD and with DTD in terms of PTSD symptoms, maternal functioning (antenatal attachment and perception of competence), reflective functions and intimate partner violence. Jittered dots represent individual data points, box plots display summary statistics, and half violins illustrate the distribution of variables. **(A)** PTSD symptoms; **(B)** antenatal attachment; **(C)** perception of maternal competence; **(D)** hypomentalizing; **(E)** disruptions in mentalizing trauma; **(F)** perpetration on intimate partner violence; **(G)** exposure to intimate partner violence during pregnancy. CM, childhood maltreatment; DTD, developmental trauma disorder; IPV, Intimate partner violence; PTSD, post-traumatic stress disorder.

ANCOVAs controlling for age and education (Table 2 and Figure 1) showed that women who experienced childhood maltreatment and met the diagnoses criteria of a DTD had poorer outcomes on all variables except intimate partner violence than women who did not experience childhood maltreatment. In contrast, women who experienced childhood maltreatment without developing a DTD differed from non-exposed women only with regard to disruptions in mentalizing trauma and adverse relationships. A non-significant trend (*p* = .008), when applying the Bonferroni correction, was also observed for PTSD symptoms. Women with DTD had more severe symptoms of PTSD, lower quality of antenatal attachment, lower perception of maternal competence, lower curiosity about and understanding of mental states, and more severe disruptions in mentalizing trauma than women who experienced childhood maltreatment without developing a DTD. No group differences were observed as regards intimate partner violence. Results controlling for the severity of exposure to maltreatment yielded the same results, except for the quality of antenatal attachment which was no longer significant following Bonferroni correction (*p* = .01; see Supplementary Table S1).

**Table 2:** ANCOVAs controlling for age and education assessing differences between participants without childhood maltreatment, and participants exposed to childhood maltreatment without DTD and with DTD in terms of PTSD symptoms, maternal functioning (antenatal attachment and perception of competence), reflective functions, and intimate partner violence.

Measures	Controls *M* (*SE*)	CM without DTD *M* (*SE*)	DTD *M* (*SE*)	*F*	*df*	*p*-value	Significant contrasts
PTSD symptoms^a^	10.04 (.73)	13.22 (1.16)	37.15 (2.27)	27.34	2, 339	< .001	C < CM without DTD^t^, DTD* CM without DTD < DTD*
Quality of antenatal attachment^a^	51.11 (.23)	50.91 (.38)	47.92 (.72)	6.54	2, 364	.002	C > DTD* CM without DTD > DTD*
Perception of maternal competence^a^	61.21 (.38)	61.73 (.62)	55.15 (1.25)	5.54	2, 354	.004	C > DTD* CM without DTD > DTD*
Hypomentalization^a^	0.19 (.02)	0.27 (.04)	0.73 (.07)	16.80	2, 359	<.001	C < DTD* CM without DTD < DTD*
Disruptions in mentalizing trauma	17.54 (.83)	25.75 (1.29)	43.03 (2.44)	53.36	2, 321	<.001	C < CM without DTD*, DTD* CM without DTD < DTD*
Intimate partner violence (Perpetration)^a^	2.24 (.26)	2.97 (.41)	4.07 (.79)	2.43	2,358	.09	N.S.
Intimate partner violence (Victimization)	1.83 (.21)	2.13 (.35)	2.81 (.66)	1.11	2,353	.33	N.S.

^a^ Nonparametric *ANCOVA* (Quade), C, controls; CM, childhood maltreatment; DTD, developmental trauma disorder; PTSD, post-traumatic stress disorder; *M*, estimated marginal average; *SE*, standard error; N.S., non significant.

A Bonferroni correction was applied to account for multiple testing (seven outcomes) and the *p*-value was fixed to.007 (.05/7).

* *p* < .007; ^t^*p* < .01.

Regarding mental health diagnoses during pregnancy, as shown in Table 3, a logistic regression controlling for age and education showed that women who had a DTD during childhood or adolescence had, respectively, 18.5-fold and 25.4-fold increased risk of suffering from a mental health disorder during pregnancy in comparison to women who had experienced maltreatment without developing a DTD (β = 2.92, *t*(1) = 22.15, *p*. < .001) and women who have not experienced maltreatment (β = 3.24, *t*(1) = 30.71, *p* < .001). Women who experienced maltreatment without developing a DTD were at similar odds of suffering from a mental health disorder during pregnancy than women without maltreatment (β = .48, *t*(1) = 2.33, *p* = .13). As shown in Table 3, the increased risk of suffering from a mental health disorder among women with DTD compared to women who experienced childhood maltreatment without developing a DTD was mainly due to a higher risk of anxiety disorders (OR = 6.53), neurodevelopmental disorders (e.g., ADHD; OR = 5.64) and personality disorders (OR = 23.75).

**Table 3:** Rates of DSM-5 mental health disorders during pregnancy among women without childhood maltreatment, and women exposed to childhood maltreatment without DTD and with DTD.

Prenatal MHD	Controls *n* = 248	CM without DTD *n* = 99	CM with DTD *n* = 26	ORs DTD vs. CM without DTD
Any diagnosis	36 (14.5%)	23 (23.2%)	22 (84.6%)	18.17*
Single disorder	24 (9.7%)	18 (18.2%)	9 (34.6%)	2.38
Comorbid disorders	12 (4.8%)	5 (5.0%)	13 (50.0%)	18.80*
Depressive disorders	2 (0.8%)	4 (4.0%)	3 (11.5%)	3.10
Bipolar disorders	1 (0.4%)	0	0	–
Anxiety disorders	17 (6.9%)	10 (10.1%)	11 (42.3%)	6.53*
Obsessive-compulsive disorders	1 (0.4%)	3 (3.0%)	3 (11.5%)	4.17
Eating disorders	3 (1.2%)	0	−0	–
Post traumatic stress disorder	2 (0.8%)	3 (3.0%)	4 (15.4%)	5.82
Neurodevelopmental disorders	9 (3.6%)	5 (5.1%)	6 (23.1%)	5.64*
Substance-related disorders	1 (0.4%)	0	2 (7.7%)	–
Personality disorder	10 (4%)	4 (4.0%)	13 (50.0%)	23.75*
Other	3 (1.2%)	0	0	–
CPTSD	–	0	9 (34.6%)	–

MHD, mental health disorder; ORs, odds ratios; CM, childhood maltreatment; DTD, developmental trauma disorder. For each column in the table, the percentages are expressed in relation to the total number of participants shown in the top row of the table.

A Bonferroni correction was applied to account for multiple testing (10 disorders) and the *p*-value was fixed to .005 (.05/10).

* *p* < .005.

Finally, as regards the continuity of complex trauma, all women who responded to the criteria of a CPTSD during pregnancy (*n* = 9, 2.4%) had presented a DTD during childhood or adolescence. Yet, the majority (*n* = 17, 65.5%,) of women with DTD did not have a CPTSD during pregnancy.

## Discussion

The study aimed to assess the frequency of probable history of DTD in a community sample of pregnant women and to examine whether women who had a DTD during childhood or adolescence differed from pregnant women who experienced childhood maltreatment without developing a DTD and from those with no history of childhood maltreatment on several markers of maternal and psychological functioning involved in the intergenerational trajectories of childhood maltreatment. We also explored the continuity of complex trauma between childhood/adolescence and adulthood. The study yielded several important and novel findings for research and practice.

*First*, we observed that 7% of pregnant women in our community sample—and 21% of women reporting a history of childhood maltreatment specifically—developed a probable DTD during childhood or adolescence. These rates appear alarming when considering the low-risk nature of our sample and the manifest effects of developmental trauma on all areas of functioning. Whereas few studies examined the prevalence of DTD in the general population, existing studies reported very similar rates of DTD (varying between 15% and 25%) in maltreated children referred to child protection agencies or for treatment (87, 88). This high level of convergence with previous childhood studies offers strong support to the validity our retrospective clinical assessment of DTD.

*Second*, we observed that women who had experienced DTD in childhood or adolescence continued to demonstrate elevated levels of distress and difficulty in adulthood, during pregnancy. Indeed, 85% of these women had a current DSM-5 disorder, which was 25.4-fold and 18.5-fold higher than pregnant women who did not experience maltreatment and women who experienced maltreatment without developing a DTD, respectively. Importantly, DTD was associated with highly complex clinical presentations in pregnant women: half of DTD women presented comorbid diagnoses in comparison to only 5% of women who experienced maltreatment but did not develop a DTD. Similarly, we also observed that pregnant women who had experienced DTD showed high levels of PTSD symptoms, important disruptions in mentalizing trauma, poor quality of antenatal bonding with the fetus, poor perception of maternal competence, and low levels of curiosity and understanding of mental states. Of note, we did not observe statistically significant group differences in terms of the perpetration or victimization of intimate partner violence, which may be due to the low variability in our low-risk sample. These results make sense given the developmental challenges posed by complex trauma. Indeed, by definition, DTD involves difficulties in most areas of development, including cognitive, affective, relational, and behavioral functioning. The extent of these difficulties may deprive children and adolescents affected by DTD of skills and opportunities that could support their recovery and resilience. Indeed, as discussed by Luyten et al. (89) and Allen (90), the profound and multifaceted challenges associated with complex trauma are likely to foster epistemic mistrust, thereby compromising youth's ability to rely on others to meaningfully reframe traumatic experiences and regain a sense of agency and control. In contrast, when trauma-related difficulties are less pervasive, certain domains of functioning may remain intact, allowing children and adolescent to preserve aspects of their self-esteem and identity and build significant relationships. Symptoms of a less extensive nature may also interfere to a lesser extent with the openness to learning from others, enabling youths to use interpersonal relationships as a secure base from which to explore and process effects of their maltreatment more effectively.

Accordingly, a *third* important finding of the current study lies in our observation that pregnant women who experienced abuse and neglect without developing a DTD during childhood or adolescence were very similar to non-exposed women in terms of psychological and maternal functioning. Indeed, they were not more likely of presenting a mental health disorder during pregnancy, had similar curiosity about and understanding of mental states, and were similarly positively engaged in motherhood, as indexed by good levels of antenatal attachment and perception of parental competence. The results are congruent with those of previous studies suggesting that most women who have experienced childhood maltreatment are resilient in that they express few internalizing or externalizing symptoms and functional impairments (58, 91). Yet, pregnant women who experienced abuse and neglect without developing a DTD still reported some disruptions in mentalizing difficult experiences, which were however much less important than those observed in women who experienced DTD. It is important to note that longitudinal studies following these women over time will be necessary to determine whether this apparent resilience remains stable postpartum. Indeed, the increased demands of motherhood and the evolving stages of the child's development may reactivate latent vulnerabilities linked to their traumatic history, potentially influencing their long-term adaptation.

*Fourth*, our findings indicate a prolonged course of complex trauma from childhood to adulthood, with approximately one-third of women with DTD experiencing CPTSD during pregnancy. Interestingly, most women who have had a DTD did not develop a CPTSD. However, they still presented significant symptoms and difficulties during pregnancy. One explanation may be that CPTSD requires the presence of clinically significant post-traumatic symptoms in addition to self-disturbance and relationship symptoms. In our sample, many women who developed a DTD did not display post-traumatic symptoms that reached the clinical threshold for a PTSD, thus explaining the discontinuity of complex trauma between childhood and adulthood in most cases. Further longitudinal studies will be needed to clarify the distinct symptoms trajectories in youth with DTD and specify the risk factors leading to CPTSD in adulthood. Nevetheless, our observation that all women having a CPTSD in the current study previously had a DTD confirm that CPTSD is an extension (into adulthood) of the childhood/adolescent diagnosis of DTD (92).

Our results have important implications for our understanding of the intergenerational trajectories of childhood maltreatment. First, the observation that most pregnant women with a history of DTD had mental health disorders during pregnancy and high levels of PTSD symptoms is alarming. Indeed, maternal psychopathology during the prenatal period is associated with poorer maternal antenatal attachment (93), poorer maternal postnatal bonding (38, 94), lower maternal sensitivity (95), negative maternal representations of the child (18), and poorer offspring development (96). We similarly observed a strong association between DTD and poor reflective functioning as well as disruptions in mentalizing trauma, which were both shown to be associated with lower maternal insighfulness and maternal sensitivity (58, 97), poorer child development (56), insecure mother-infant attachment relationships (60, 77, 98), controlling and unresponsive maternal behaviors (99, 100), as well as higher risk of intergenerational cycles of child abuse (62). Finally, our observation that DTD was associated with poor representations of the child-to-be (indexed by low scores on the quality of attachment scale of the MAAS) and negative representations of the self as a mother (indexed by low levels of perception of maternal competence) is likely to be important for postnatal maternal bonding and offspring development given the well-documented importance of maternal representations in shaping infant development and functioning (101).

In sum, our findings underscore the heterogeneity of developmental trajectories in the face of abuse or neglect and nuance conclusions from previous studies suggesting that exposure to abuse or neglect impairs maternal well-being and functioning during pregnancy (15, 102, 103). Our findings that DTD, but not exposure to maltreatment alone, is a risk factor for psychological and maternal functioning in pregnant women call for future longitudinal and multimodal studies aimed at assessing the impact of a history of DTD on pregnancy outcomes, offspring development, maternal behaviors, and intergenerational cycles of childhood maltreatment.

### Strenghts and limitations

Our study has several strengths. First, we assessed complex trauma using well-characterized and recognized diagnostic criteria that were evaluated by two independent clinical psychologists using structured clinical interviews, whereas most previous studies on DTD relied on diagnostic algorithms derived from self-reported questionnaires for which construct validity remains to be confirmed. Second, our sample was rather large (*n* = 373) for a multimodal study including blind diagnostic assessment, and included non-maltreated women, women who experienced maltreatment without developing a DTD, and women with a history of DTD, which allowed us to examine group differences. Third, our analyses controlled for relevant confounding variables, including the severity of exposure to childhood maltreatment. Our study however has several limitations. One limitation was that our sample was rather homogenous in terms of racial/ethnic backgrounds, which limits the generalizability of the results. Secondly, despite the quality of our assessment of complex trauma in comparison to most prior study, DTD remained assessed retrospectively using structured clinical interviews (SCID-5) that were not specifically designed to enquire about complex trauma. Future longitudinal study beginning during childhood and using the most recent assessment procedures are required. In addition, we cannot exclude a negative recall bias in participants with current psychological distress, which may have contributed to higher retrospective reports of symptoms related to DTD in women with current mental health disorders (104). Fourthly, the small number of participants with current CPTSD limited statistical analyses on this specific subgroup. Future studies should examine the trajectory between DTD and CPTSD in more details. Fifhtly, whilst the inclusion of intimate partner violence as a dependent variable in the study is a strength, the analysis was limited to violence occurring during pregnancy. It is possible that different or stronger associations with our primary variable of interest—DTD—might emerge at other stages of life. Finally, several factors were not controlled for, including the age at onset of maltreatment, types of childhood maltreatment, chronicity of abuse and neglect, trauma exposure during adulthood, therapeutic and pharmacological treatments, and resilience-promoting factors at the community, interpersonal, sociodemographic, and intraindividual levels.

### Clinical implications

The findings have several clinical implications. First, if one aims for early identification of women at-risk of presenting significant psychological difficulties during their pregnancy, a target screening of previous developmental trauma may be more cost-effective than a larger screening of exposure to abuse or neglect. Second, the findings call for prenatal or early trauma-informed and trauma-focused interventions for mothers who have experienced developmental trauma aiming at mitigating the intergenerational effects of abuse and neglect and their impact on women's health and functioning. This includes programs such as *STEP: Supporting the Transition to and Engagement in Parenthood* (105, 106), a mentalization-based group intervention for pregnant women who experienced childhood maltreatment, the *Perinatal Child-Parent Psychotherapy* (107), a psychotherapy for pregnant women who experienced abuse or domestic violence aiming to reduce the burden of parental traumatic experiences, or *Mom Power* (108, 109), a community-based multifamily parenting intervention for high-risk, trauma-exposed mothers of young children. Third, the findings provide further evidence in support of considering DTD as a distinct disorder in psychiatric nosology (63, 92, 110, 111). Indeed, this diagnosis seems to enable the identification of a specific group of women who present a distinct developmental trajectory and are especially at risk.

## Data Availability

The raw data supporting the conclusions of this article will be made available by the authors, without undue reservation.
