# Supplementary material for: Psychological functioning in pregnant women who experienced complex trauma
Source: Front Glob Womens Health. 2025 Jul 31;6:1611034. doi: 10.3389/fgwh.2025.1611034 (PMC12350466; doi:10.3389/fgwh.2025.1611034)
Supplement: Supplementary file 1 [file Table1.docx]

**ELECTRONIC SUPPLEMENT**

**Table S1**

*ANCOVAs controlling for age, education and severity of maltreatment assessing differences between participants exposed to childhood maltreatment without DTD and with DTD in terms of PTSD symptoms, maternal functioning (antenatal attachment and perception of competence), intimate partner violence and reflective functions*

| **Measures** | **CM without DTD**  *M* (*SE*) | **CM with DTD**  *M*(*SE*) | ***F*** | ***df*** | ***p* value** |
| --- | --- | --- | --- | --- | --- |
| PTSD symptoms ^a^ | 14.20 (1.27) | 37.98(2.59) | 37.94 | 1, 106 | <.001 |
| Quality of antenatal attachment ^a^ | 50.89 (.42) | 48.03 (.84) | 6.53 | 1, 112 | .012 |
| Perception of maternal competence ^a^ | 62.13 (.61) | 55.98 (1.29) | 8.43 | 1, 107 | .004 |
| Hypomentalization ^a^ | 0.30 (.04) | .77 (.09) | 18.52 | 1, 112 | <.001 |
| Disruptions in mentalizing trauma | 27.07 (1.16) | 44.02 (2.30) | 41.76 | 1, 103 | <.001 |
| Intimate partner violence (Perpetration)^a^ | 3.18 (.51) | 4.14 (1.01) | .10 | 1,110 | .76 |
| Intimate partner violence (Victimization) | 2.25 (.31) | 2.20 (.62) | .01 | 1,106 | .94 |

*Note.* ^a^ Nonparametric *ANCOVA* (Quade), CM = Childhood maltreatment, DTD = Developmental trauma disorder, *M* = Estimated marginal average, *SE* = Standard error, PTSD = Post-traumatic stress disorder. A Bonferroni correction was applied to account for multiple testing and the *p*-value was fixed to .007 (.05/7).*p* < .007
